# Expression of D-Amino Acid Oxidase (*DAO*/*DAAO*) and D-Amino Acid Oxidase Activator (*DAOA/G72*) during Development and Aging in the Human Post-mortem Brain

**DOI:** 10.3389/fnana.2017.00031

**Published:** 2017-04-06

**Authors:** Vinita Jagannath, Zoya Marinova, Camelia-Maria Monoranu, Susanne Walitza, Edna Grünblatt

**Affiliations:** ^1^Molecular and Neurobiochemistry Laboratory, Centre for Child and Adolescent Psychiatry Research, Department of Child and Adolescent Psychiatry and Psychotherapy, University Hospital of Psychiatry Zurich, University of ZurichZurich, Switzerland; ^2^Department of Neuropathology, Institute of Pathology, University of WürzburgWürzburg, Germany; ^3^Neuroscience Center Zurich, University of Zurich and ETH ZurichZurich, Switzerland; ^4^Zurich Center for Integrative Human Physiology, University of ZurichZurich, Switzerland

**Keywords:** DAO/DAAO, DAOA/G72, gene and protein expression, DNA methylation, human post-mortem brain regions

## Abstract

In the brain, D-amino acid oxidase (DAO/DAAO) mainly oxidizes D-serine, a co-agonist of the *N*-methyl-D-aspartate (NMDA) receptors. Thus, DAO can regulate the function of NMDA receptors via D-serine breakdown. Furthermore, DAO activator (DAOA)/G72 has been reported as both DAOA and repressor. The co-expression of DAO and DAOA genes and proteins in the human brain is not yet elucidated. The aim of this study was to understand the regional and age span distribution of DAO and DAOA (mRNA and protein) in a concomitant manner. We determined DAO and DAOA mRNA and protein expression across six brain regions in normal human post-mortem brain samples (16 weeks of gestation to 91 years) using quantitative real-time reverse transcription-polymerase chain reaction and enzyme-linked immunosorbent assay. We found higher expression of *DAO* mRNA in the cerebellum, whereas lower expression of DAO protein in the cerebellum compared to the other brain regions studied, which suggests post-transcriptional regulation. We detected DAOA protein but not *DAOA* mRNA in all brain regions studied, suggesting a tightly regulated expression. To understand this regulation at the transcriptional level, we analyzed DNA methylation levels at *DAO* and *DAOA* CpG sites in the cerebellum and frontal cortex of control human post-mortem brain obtained from Gene Expression Omnibus datasets. Indeed, *DAO* and *DAOA* CpG sites in the cerebellum were significantly more methylated than those in the frontal cortex. While investigating lifespan effects, we found that *DAO* mRNA levels were positively correlated with age <2 years in the cerebellum and amygdala. We also detected a significant positive correlation (controlled for age) between DAO and DAOA protein in all of the brain regions studied except for the frontal cortex. In summary, DAO and DAOA expression in the human brain are both age and brain region dependent.

## Introduction

Brain development is a continuous process which extends from the prenatal period into adulthood (Rapoport et al., 2012). Abnormal neurodevelopmental processes are implicated in many psychiatric disorders such as schizophrenia, attention deficit hyperactivity disorder, autism spectrum disorder, and intellectual disability (Rapoport et al., 2012; Miller et al., 2016). Moreover, altered gene expression across different brain regions and lifespan has been reported in psychiatric disorders (Sibille, 2013; Darby et al., 2016). For example, schizophrenia risk genes are reported to be associated with transcripts which are either enriched in specific brain regions or unique to the human brain, and some also show preferential expression in the fetal brain (Kang et al., 2011; Kleinman et al., 2011). Therefore, it is important to analyze the brain region-specific expression of risk genes for psychiatric disorders in the normal human brain across the lifespan to understand the mechanisms underlying pathological changes in psychiatric disorders.

D-amino acid oxidase (DAO/DAAO; from now on addressed as DAO) is a flavoenzyme, which oxidizes D-amino acids. Its main substrate in the brain is D-serine (Pollegioni et al., 2007; Sacchi et al., 2012). D-serine is a co-agonist of *N*-methyl-D-aspartate (NMDA) receptors. In addition to glutamate, NMDA receptors require a co-agonist (glycine or D-serine) binding at the glycine modulatory site in order to function normally. D-serine is shown to be more potent in binding to NMDA receptors than glycine (Matsui et al., 1995; Shleper et al., 2005; Sacchi et al., 2012). Thus, DAO can regulate the function of NMDA receptors via D-serine breakdown, which may lead to NMDA receptor hypofunction (Kantrowitz and Javitt, 2010; Labrie and Roder, 2010; Verrall et al., 2010; Labrie et al., 2012). One of the possible explanations for NMDA receptor hypofunction theory proposed in schizophrenia is probably an increased activity of DAO leading to decreased D-serine. DAO activator (DAOA)/G72 (from now on addressed as DAOA) binds to DAO, but the effect of DAOA on DAO is controversial. This is because DAOA is reported to both increase (Chumakov et al., 2002; Chang et al., 2014) and decrease (Sacchi et al., 2008, 2011) the activity of DAO. DAOA is localized in mitochondria and reported to modulate its function (Kvajo et al., 2008; Sacchi et al., 2011; Otte et al., 2014). Thus, the exact function of DAOA is not yet completely understood.

The human *DAO* gene located on chromosome 12q24 encodes for a ∼39 kDa protein (Almond et al., 2006; Sacchi et al., 2008; Verrall et al., 2010) and the human *DAOA* gene encodes for a ∼20 kDa protein (Benzel et al., 2008). The genes *DAOA*/*G30* overlap and are transcribed from opposite strands on chromosome 13q33. The polymorphisms of these genes in non-coding regions have been described to be associated with the pathophysiology of schizophrenia and bipolar disorder (Detera-Wadleigh and McMahon, 2006; Korostishevsky et al., 2006; Corvin et al., 2007; Prata et al., 2008; Mossner et al., 2010; Labrie et al., 2012; Gatt et al., 2015; Liu et al., 2016). However, a recent genome-wide association study conducted by the Psychiatric Genomics Consortium found that out of 108 schizophrenia-associated loci, none were within *DAO* and *DAOA* gene regions (Schizophrenia Working Group of the Psychiatric Genomics Consortium, 2014). There are several lines of evidence showing regional and cellular expression of DAO mRNA and protein in the human brain (Kapoor et al., 2006; Verrall et al., 2007; Habl et al., 2009; Ono et al., 2009), however, none of these studies isolated mRNA and protein concomitantly from the same brain tissue as we did in this study. On the other hand, the reports of DAOA mRNA and protein expression in the human brain remain unconvincing to date. Studies showing *DAOA* mRNA expression in the human brain are limited (Chumakov et al., 2002; Korostishevsky et al., 2006). Moreover, the expression of DAOA mRNA and protein in the human brain has been questioned (Benzel et al., 2008; GTEx Consortium, 2015). The human protein atlas detected DAOA protein (also known as pLG72, from now on addressed as DAOA) in the cerebellum and the cerebral cortex (Uhlen et al., 2015). Thus, the regulation of DAO and DAOA expression in the human brain has not yet been elucidated.

Furthermore, since DNA methylation can lead to both increases and decreases in gene expression (Wagner et al., 2014), it may play a crucial role in the regulation of normal brain development (Fan et al., 2001). Hannon et al. (2015) quantified DNA methylation using Illumina 450K array in normal human post-mortem brain, and reported that DNA methylation levels in cortical regions differ from the cerebellum. Moreover, epigenetic regulation of gene expression is very important in the human brain (Graff et al., 2011; Numata et al., 2012). However, the extent to which DNA methylation or other epigenetic modifications affect the expression of genes important for human brain function, such as *DAO*/*DAOA*, is largely unknown.

In the present study, our main aim was to understand concomitant DAO and DAOA mRNA and protein expression in the normal human post-mortem brain during development and aging in six brain regions. We also correlated *DAO* mRNA and DAO protein as well as DAO protein and DAOA protein to understand their concomitant expression in different brain regions. Furthermore, we assessed the potential effect of *DAO* and *DAOA* polymorphisms on DAO and DAOA expression levels to elucidate their role in the regulation of DAO and DAOA expression. To understand the hypothesized regulation of DAO and DAOA expression at the transcription level in an indirect manner, we determined the DNA methylation levels at CpG sites of *DAO* and *DAOA* genes in the cerebellum and the frontal cortex of control human post-mortem brain obtained from Gene Expression Omnibus (GEO) datasets.

## Materials and Methods

### Subjects

Human post-mortem brain samples from six brain regions were studied. These brain samples were from 55 subjects with age ranging from 16 weeks of gestation to 91 years, who had no history of psychiatric or neurological illness, and their post-mortem brain samples showed no neuropathological evidence of any neuropsychiatric disorder. The brain samples were collected from six brain regions, namely: the cerebellum (*n* = 47), brainstem (*n* = 49), amygdala (*n* = 47), striatum (*n* = 52), thalamus (*n* = 52), and frontal cortex (*n* = 54). These brain samples were procured from the Department of Neuropathology, Institute of Pathology, University of Würzburg, Germany (member of the BrainNet Europe-BNEII) and the London Neurodegenerative Diseases Brain Bank, UK. Informed written consent for tissue donation was obtained from the individuals or the next of kin. The causes of death of the study population are presented in Supplementary Table S6. This study was approved by the Cantonal Ethic Commission of Zurich (Ref. Nr. EK: KEK-ZH-Nr. 2013-0177).

### DNA, RNA, and Protein Isolation

DNA, RNA, and protein were simultaneously isolated from the same frozen human post-mortem brain samples using AllPrep DNA/RNA/Protein Mini Kit (Qiagen, Switzerland). Firstly, frozen brain tissue samples weighing around 27–34 mg were disrupted and homogenized in the buffer provided in the kit using the TissueLyser II (Qiagen, Switzerland). Secondly, tissue lysates were added to the AllPrep DNA spin column, and DNA was eluted. Thirdly, RNA was eluted after adding an ethanol treated sample to a RNeasy spin column. Finally, proteins in the sample were precipitated and pelleted by centrifugation. Since the buffer provided in the kit interferes with protein determination and does not dissolve proteins completely, protein eluates were acetone precipitated, and precipitated proteins were redissolved in urea buffer (7 M urea, 2 M thiourea, 2% CHAPS, and trace bromophenol, pH 8).

### TaqMan SNP Genotyping

DNA isolated from post-mortem human cerebellum (*n* = 46), striatum (*n* = 2), thalamus (*n* = 5), and amygdala (*n* = 2) were used for genotyping. DNA concentrations, A260/A280, and A260/A230 ratios were measured using a spectrophotometer (NanoVue Plus, GE Healthcare Life Sciences). Real-time polymerase chain reaction (PCR) was used to genotype *DAO* (rs3918347, rs4623951), and *DAOA* (rs778293, rs3916971, rs746187) polymorphisms. These single-nucleotide polymorphisms (SNPs) were chosen based on previously reported association with schizophrenia (Allen et al., 2008). DNA, TaqMan^^®^^ Genotyping Master Mix (Applied Biosystems, USA), and SNP Genotyping Assays (rs778293 assay number: C_8704507_10; rs3916971 assay number: C_27495752_10; rs746187 assay number: C_1925241_10; rs3918347 assay number: C_27937201_10; and rs4623951 assay number: C_32177440_10, all from Applied Biosystems, USA) were combined in a 384-well plate. Real-time PCR was performed in a C1000^TM^ CFX384^TM^ Thermal cycler (Bio-Rad) using TaqMan^^®^^ SNP Genotyping Assay PCR standard protocol. Genotypes were determined by the allelic discrimination program of Bio-Rad CFX Manager^TM^ Software version 2.1. The Hardy–Weinberg equilibrium (HWE) *p*-value and allele frequencies were computed using PLINK software (Purcell et al., 2007), and *p* < 0.05 was considered as statistically significant.

### Quantification of *DAO* and *DAOA* mRNA Levels Using qRT-PCR

RNA isolated from post-mortem human cerebellum (*n* = 44), brainstem (*n* = 40), amygdala (*n* = 39), striatum (*n* = 35), thalamus (*n* = 37), and frontal cortex (*n* = 36) were used for quantitative real-time reverse transcription-PCR (qRT-PCR). A spectrophotometer (NanoVue Plus, GE Healthcare Life Sciences) was used to measure RNA concentrations, A260/A280, and A260/A230 ratios. RNA integrity was analyzed using Experion automated electrophoresis system (Bio-Rad, Switzerland) in a subset of samples. RNA (500 ng) was reverse transcribed using iScript^TM^ cDNA Synthesis Kit (Bio-Rad, Switzerland) as per manufacturer’s protocol. In a subset of samples, negative controls were prepared with RNA without reverse transcriptase enzyme. qRT-PCR was performed using cDNA, QuantiFast SYBR Green PCR kit (Qiagen, Switzerland), 1 μM forward and reverse DAO primers (Microsynth, Switzerland), and reference genes [β-actin (*ACTB*) (QT01680476), aminolevulinate synthetase (*ALAS1*) (QT00073122), ribosomal protein L13a (*RPL13A*) (QT00089915), alanyl-tRNA synthetase (*AARS*) (QT00054747), glyceraldehyde-3-phosphate dehydrogenase (*GAPDH*) (QT01192646), peptidylprolyl isomerase A (*PPIA*) (QT00866137), ribosomal RNA (*R18S*) (QT00019936), and X-prolyl aminopeptidase 1 (*XPNPEP1*) (QT00051471); all from Qiagen, Switzerland]. These eight reference genes were selected based on previous reports of their stability in human post-mortem brain (Durrenberger et al., 2012). The DAO primers used in this study have been described by Verrall et al. (2007) (forward primer: CGCAGACGTGATTGTCAACT; reverse primer: GGATGATGTACGGGGAATTG). *DAO* mRNA levels were normalized to the reference genes. PCR efficiencies were calculated using LinRegPCR program (Ruijter et al., 2009) and mean PCR efficiencies for all studied amplicons were found to be between 88 and 95%. Normalized *DAO* mRNA levels were quantified by importing qRT-PCR quantification cycle (Cq) values of the gene of interest and reference genes across brain regions and lifespan into qBASE plus software (Biogazelle, Belgium) which utilizes gene-specific amplification efficiencies, and allows normalization with multiple reference genes. The qBASE plus software automatically selects multiple stably expressed reference genes in the samples across brain regions and lifespan by taking into account their stability. The software carries out a normalization of the gene of interest with multiple reference genes, ultimately producing calibrated normalized relative quantities of the gene of interest which was used to perform statistical analysis (Hellemans et al., 2007). To detect *DAOA* mRNA in human post-mortem brain, qRT-PCR was performed using several different primers for *DAOA* gene described by Benzel et al. (2008), Cheng et al. (2014), and pre-designed primers [QT00058863 (Qiagen), Hs.PT.58.555086 (IDT), 4331182 (Thermo Fisher Scientific), qHsaCEP0024792 (Bio-Rad)]. As a positive control, these primers were also tested on the cDNA prepared from human neuroblastoma cells (SK-N-SH and SH-SY5Y) overexpressing DAOA, and these primers detected *DAOA* mRNA in these cells, but not in the human post-mortem brain samples (data not shown). *DAOA* mRNA was still undetectable in human post-mortem brain after increasing the cDNA amounts in qRT-PCR. Furthermore, amplification using QuantiTect Whole Transcriptome Kit (207043, Qiagen) followed by qRT-PCR to detect *DAOA* mRNA levels was not successful. In summary, we were unable to quantify *DAOA* mRNA levels with any of the aforementioned methods in human post-mortem brain.

### Quantification of DAO and DAOA Protein Levels Using Commercially Available ELISA Kits

Protein isolated and acetone-precipitated from post-mortem human cerebellum (*n* = 40), brainstem (*n* = 47), amygdala (*n* = 46), striatum (*n* = 48), thalamus (*n* = 48), and frontal cortex (*n* = 54) were used for enzyme-linked immunosorbent assay (ELISA). Total protein concentration was quantified using the Bradford assay (Sigma-Aldrich; Bradford, 1976). DAO and DAOA protein concentrations were quantified using a commercially available DAO ELISA kit (SEJ298Hu; Cloud-Clone Corp.) with a high specificity and a sensitivity of 0.56 ng/mL and a commercially available DAOA ELISA kit (SEJ297Hu; Cloud-Clone Corp.) with a high specificity and a sensitivity of 0.061 ng/mL according to the manufacturer’s instructions. A standard curve was obtained by plotting absorbance versus different concentration of protein standards provided with the kit, and DAO and DAOA protein concentrations were determined from the standard curve. Normalized DAO and DAOA protein concentrations were calculated by dividing DAO and DAOA protein concentrations by the total protein concentration measured using Bradford assay. The specificity of DAO and DAOA ELISA kits was proved using sodium dodecyl sulfate polyacrylamide gel electrophoresis and Western blotting which is described in detail in Supplementary Methods S1.

### DNA Methylation Levels across *DAO* and *DAOA* CpG Sites in the Cerebellum and Frontal Cortex

The processed and normalized methylation beta values from two datasets: GEO accession number GSE61431 as described in the study of Pidsley et al. (2014) and GSE63347 as described by Horvath et al. (2015) were downloaded from the GEO website^1^. From these datasets, the methylation beta values for *DAO* and *DAOA* CpG sites from the cerebellum and frontal cortex of controls were extracted. *DAO* (cg03868278, cg22621535, cg22801690) and *DAOA* (cg00809502) CpG sites in the repeat masker region sites were excluded from the analysis. *DAO* CpG sites: cg01694331, cg25362648, cg18037826, cg12592321 lie in the exon 1 and cg26725638 in the exon 2 of *DAO* gene. *DAOA* CpG sites: cg22773522, cg25623522, cg01297020 are in the promoter region, cg13846327 and cg20888753 in the intron 2, and cg11374446 in the intron 3 region of the *DAOA* gene. In the GSE61431 dataset, there were 23 control cerebellum and frontal cortex samples ranging from ages 25 to 96 years. In the GSE63347 dataset, there were nine control cerebellum samples in the ages between 38 and 64 years and 17 control frontal cortex samples between ages 32 and 64 years.

### Statistical Analysis

IBM^^®^^ SPSS^^®^^ Statistics (version 21) software was used for statistical analysis. Shapiro–Wilk test with Lilliefors significance correction was used to assess the normality of the distribution of mRNA, protein expression, and DNA methylation data. *DAO* mRNA, DAO and DAOA protein expression, and DNA methylation data showed both normal and non-normal distribution across different brain regions. In order to maintain consistency between statistical evaluations, non-parametric tests were used even for normally distributed data. Linear regression with brain regions as dummy variables was used after a logarithmic transformation of the non-normally distributed data to determine the differences in *DAO* mRNA, DAO and DAOA protein expression in the brain regions studied; *p* < 0.05 was taken as statistically significant. The differences in *DAO* mRNA, and DAO and DAOA protein levels across different age groups were assessed by the Kruskal–Wallis test followed by pair-wise comparisons with the Mann–Whitney test by adjusting *p*-value based on the number of comparisons (Bonferroni correction, *p* < 0.005). As we do not have post-mortem brain samples from ages 2 to 20 years, we divided the ages for correlation into two groups, namely less than 2 years and more than 20 years. Then, Spearman’s rank correlation test was used to assess the correlation between age groups (<2 years and >20 years) and *DAO* mRNA, DAO and DAOA protein levels; *p* < 0.05 was taken as statistically significant. We correlated *DAO* mRNA with DAO protein levels, and DAO protein levels with DAOA protein levels across six brain regions by controlling for age using partial correlation, *p* < 0.05 was taken as statistically significant. To analyze the effect of *DAO* and *DAOA* SNP genotypes on *DAO* mRNA, DAO and DAOA protein expression, analysis of covariance (ANCOVA) with age as a covariate was used after a logarithmic transformation of not normally distributed *DAO* mRNA and DAOA protein expression data and the inverse transformation of the non-normally distributed DAO protein expression data; *p* < 0.05 was taken as statistically significant. The differences in methylation levels between the cerebellum and frontal cortex at each CpG site was assessed using the Mann–Whitney test by adjusting *p*-value based on a number of CpG sites analyzed (Bonferroni correction, *p* < 0.005). The correlation between age and methylation levels at each CpG site in the cerebellum and frontal cortex was assessed using Spearman’s rank correlation test, and *p* < 0.05 was considered statistically significant. GraphPad Prism software (version 6.01) was used to plot the graphs. The *post hoc* power analyses for *DAO* mRNA, DAO and DAOA protein expression across five age groups were conducted using G*Power software (Faul et al., 2009), the effect sizes were determined from means of age groups, and the alpha level was set at 0.05 (Supplementary Table S7).

## Results

### DAO and DAOA Expression in Different Brain Regions

The current study aimed to assess brain region-specific DAO and DAOA expression across the lifespan in normal human post-mortem brain using qRT-PCR and ELISA techniques. The human post-mortem brain samples were obtained from 55 subjects with no clinical or neuropathological evidence of neuropsychiatric disorders. The demographic characteristics of the study sample across five age groups are presented in Supplementary Table S1. In order to assess any confounding factors inherent in human post-mortem studies, we addressed factors such as post-mortem interval (PMI) and gender. The length of PMI did not differ significantly between different age groups as assessed by the Kruskal–Wallis test. Gender had a significant effect on *DAO* mRNA (*p* = 0.048) and protein (*p* = 0.047) expression in the thalamus and frontal cortex, respectively (Mann–Whitney test, *p* < 0.05). *DAO* mRNA in the thalamus and DAO protein in the frontal cortex was significantly more expressed in males than in females. Gender had no significant effect on DAOA protein expression in all brain areas studied. There was no statistically significant correlation between PMI and *DAO* mRNA, DAO and DAOA protein levels in the studied brain regions (Spearman’s rank correlation, *p* > 0.05).

**FIGURE 1:**
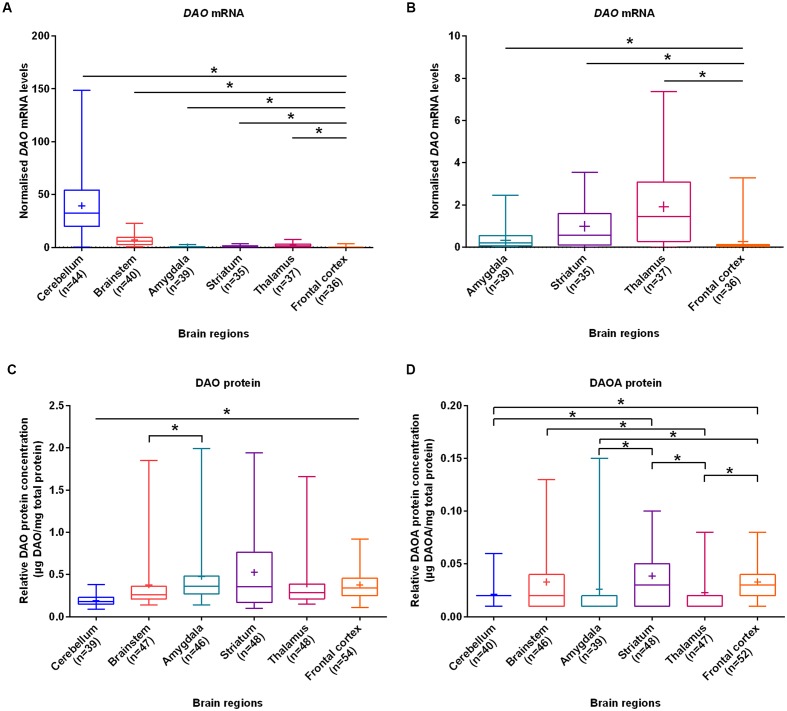
***DAO* mRNA, DAO and DAOA protein levels quantified in six regions of human post-mortem brain. (A)** Normalized *DAO* mRNA levels across six brain regions. **(B)** For clarity, this figure focuses only on amygdala, striatum, thalamus, and frontal cortex results presented in panel **(A)**. DAO protein levels (μg DAO/mg total protein) **(C)** and DAOA protein levels (μg DAOA/mg total protein) **(D)** in studied brain regions. Values are presented as box and whisker plots, whiskers represent minimum to maximum values, “+” indicates mean values, and ^∗^*p* < 0.05. Linear regression with brain regions as dummy variables was used after logarithmic transformation of non-normally distributed data.

With the aim of investigating brain region-specific patterns of DAO and DAOA, we assessed their expression in the normal brain. We found that *DAO* mRNA was significantly more expressed in the cerebellum (set to 100%), followed by the brainstem (17.6%), thalamus (4.8%), striatum (2.5%), amygdala (0.9%), and the frontal cortex (0.7%) as assessed by linear regression (**Figures 1A,B**). In contrast to *DAO* mRNA, DAO protein was significantly less expressed in the cerebellum than in the remaining brain regions. In the amygdala (set to 100%), DAO protein was significantly more expressed than in the brainstem (79.2%; **Figure 1C**). DAOA protein was significantly more expressed in the frontal cortex (set to 100%) than in the cerebellum (65.2%), amygdala (80.4%), and the thalamus (69.6%). In the striatum (set to 100%), DAOA protein was significantly more expressed than in the amygdala (66.7%) and the cerebellum (54.1%). DAOA protein was significantly less expressed in the thalamus (57.7%) than in the brainstem (85.6%) and striatum (set to 100%; **Figure 1D**). Thus, DAO and DAOA exhibit region-specific expression patterns in the human brain.

### Expression of *DAO* mRNA, DAO and DAOA Protein across Lifespan in Six Brain Regions

The expression patterns across lifespan were analyzed using two approaches. The first approach was by grouping ages into five groups, while the second approach was by correlating ages with expression levels which provided information on expression patterns during brain development, maturation, and degeneration. We assessed DAO and DAOA expression across age groups in six brain regions to understand critical and sensitive periods during brain development. As the sample size in each age group was small, we performed a power analysis which showed reasonable power (>0.76) in the cerebellum, striatum, and thalamus for *DAO* mRNA as well as reasonable power (>0.64) in the cerebellum, striatum, and frontal cortex for DAO protein, but the power was too low in the brain regions studied for DAOA protein to conduct statistical analysis (Supplementary Table S7).

**FIGURE 2:**
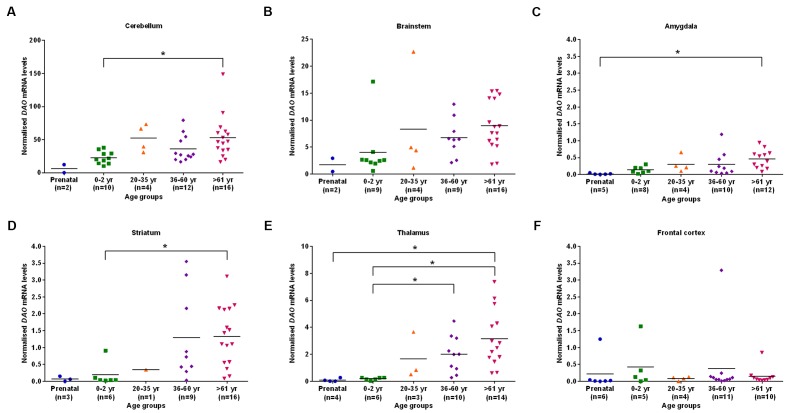
***DAO* mRNA levels across five different age groups in six regions of human post-mortem brain (A–F).** Data presented as scatter plots. Kruskal–Wallis test followed by pairwise comparisons with the Mann–Whitney test (^∗^*p* < 0.005) was performed to assess the differences in *DAO* mRNA levels between different age groups across six brain regions.

We found statistically significant (*p* < 0.05) differences in *DAO* mRNA levels across the five different age groups, as assessed by the Kruskal–Wallis test, in the cerebellum, brainstem (**Figure 2B**), amygdala, striatum, and thalamus. However, differences in *DAO* mRNA levels were found to be statistically non-significant in the frontal cortex (**Figure 2F**). Pair-wise comparisons using the Mann–Whitney test showed statistically significant differences (*p* < 0.005) in *DAO* mRNA levels between age groups 0–2 years and >61 years in the cerebellum (**Figure 2A**) and striatum (**Figure 2D**), between prenatal and >61 years in the amygdala (**Figure 2C**), and between prenatal and >61 years, 0–2 years and 36–60 years, 0–2 years and >61 years in the thalamus (**Figure 2E**).

**FIGURE 3:**
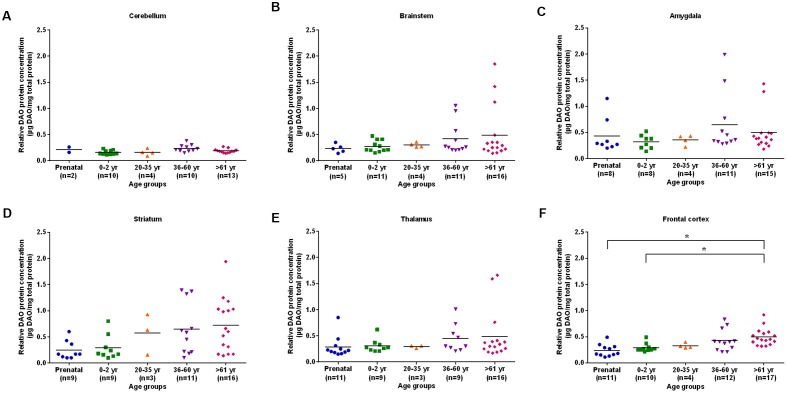
**DAO protein levels (μg DAO/mg total protein) across different age groups in six regions of human post-mortem brain (A–F).** Data presented as scatter plots. Differences in DAO protein levels across different age groups was assessed by the Kruskal–Wallis test followed by pairwise comparisons with the Mann–Whitney test (^∗^*p* < 0.005).

**FIGURE 4:**
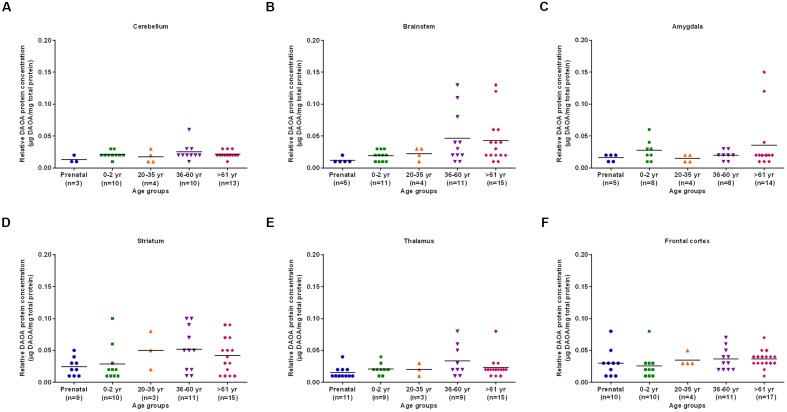
**DAOA protein expression (μg DAOA/mg total protein) across five different age groups in six regions of human post-mortem brain (A–F).** Values are presented as scatter plots. Differences in DAOA protein levels across different age groups were assessed with the Kruskal–Wallis test.

The differences in DAO protein levels across the five different age groups was found to be statistically significant (*p* < 0.05), as assessed by the Kruskal–Wallis test, in the cerebellum (**Figure 3A**), striatum (**Figure 3D**), and frontal cortex (**Figure 3F**). However, differences in DAO protein levels were found to be statistically non-significant in the brainstem (**Figure 3B**), amygdala (**Figure 3C**), and thalamus (**Figure 3E**). Pair-wise comparisons with the Mann–Whitney test showed statistically significant differences (*p* < 0.005) in DAO protein levels for age groups >61 years versus prenatal and 0–2 years in the frontal cortex (**Figure 3F**). The differences in DAOA protein levels across the five different age groups were found to be statistically significant (*p* < 0.05) in the brainstem as assessed by the Kruskal–Wallis test. There were no statistically significant differences in DAOA protein levels in the cerebellum, amygdala, striatum, thalamus, and frontal cortex (**Figure 4**). The specificity of DAO and DAOA ELISA kits was demonstrated by Western blot as presented in Supplementary Figure S2.

Using the second approach, we correlated age (<2 years and >20 years) with DAO and DAOA expression across different brain regions to understand the critical developmental period and brain region in DAO and DAOA expression. We found a statistically significant positive correlation (*p* < 0.05) as assessed by Spearman’s rank correlation test, between age less than 2 years and *DAO* mRNA levels in the cerebellum and amygdala (**Table 1**). Furthermore, there was a significant positive correlation (Spearman’s rank correlation, *p* < 0.01) between age more than 20 years and DAO protein concentration in the frontal cortex (**Table 1**). There was no statistically significant correlation between age and DAOA protein concentration in the brain regions studied (**Table 1**).

**Table 1:** Correlation between age and *DAO* mRNA, DAO and DAOA protein expression across brain regions of human post-mortem brain.

	Brain regions	Age in years					
		Prenatal and 0–2 years			20–91 years		
		*N*	Spearman’s rank correlation co-efficient	*p*-value	*N*	Spearman’s rank correlation co-efficient	*p*-value
Normalized *DAO* mRNA levels	Cerebellum	12	0.828	0.001^∗∗^	32	0.127	0.488
	Brainstem	11	0.310	0.354	29	0.117	0.546
	Amygdala	13	0.669	0.017^∗^	26	0.261	0.198
	Striatum	9	0.008	0.983	26	0.259	0.202
	Thalamus	10	0.427	0.218	27	0.254	0.200
	Frontal cortex	11	0.352	0.288	25	0.022	0.918

Relative DAO protein concentration (μg DAO/mg total protein)	Cerebellum	12	-0.495	0.102	27	-0.143	0.478
	Brainstem	16	0.092	0.736	31	0.077	0.679
	Amygdala	16	-0.122	0.652	30	0.162	0.392
	Striatum	18	0.082	0.747	30	0.142	0.455
	Thalamus	20	0.285	0.224	28	0.180	0.359
	Frontal cortex	21	0.185	0.422	33	0.482	0.004^∗^

Relative DAOA protein concentration (μg DAOA/mg total protein)	Cerebellum	13	0.120	0.695	27	0.099	0.623
	Brainstem	16	0.332	0.210	30	0.324	0.081
	Amygdala	13	0.318	0.268	26	0.306	0.121
	Striatum	19	0.037	0.879	29	-0.120	0.536
	Thalamus	20	0.195	0.411	27	0.021	0.916
	Frontal cortex	20	-0.200	0.398	32	0.149	0.417

^∗^*p < 0.05; ^∗∗^*p* < 0.01*.

### Correlation between *DAO* mRNA, DAO and DAOA Protein in Different Brain Regions

In order to assess whether there is a concomitant expression of *DAO* mRNA and its protein, we correlated the two in various brain regions. We did not find any statistically significant correlation (partial correlation controlled for age) between *DAO* mRNA and DAO protein in each of the studied brain regions (Supplementary Figure S3).

**FIGURE 5:**
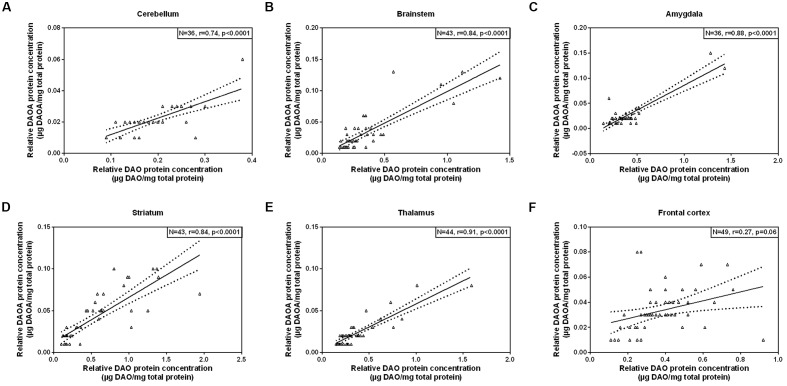
**Correlation between DAO (μg DAO/mg total protein) and DAOA protein expression (μg DAOA/mg total protein) in six regions of the human post-mortem brain (A–F).** Data is presented as scatter plots with linear fit and 95% confidence intervals. Correlation between DAO and DAOA protein levels in different brain regions were assessed with the partial correlation test, controlled for age; *p* < 0.05 was taken as statistically significant (*N*: sample size, *r*: correlation co-efficient).

As we concomitantly measured DAO and DAOA proteins in the human brain, we examined whether these two proteins correlate in each brain region. A statistically significant positive correlation (partial correlation controlled for age) was found between DAO and DAOA protein expression in the cerebellum [*r*(36) = 0.74, *p* < 0.0001; **Figure 5A**], the brainstem [*r*(43) = 0.84, *p* < 0.0001; **Figure 5B**], the amygdala [*r*(36) = 0.88, *p* < 0.0001; **Figure 5C**], the striatum [*r*(43) = 0.84, *p* < 0.0001; **Figure 5D**], and the thalamus [*r*(44) = 0.91, *p* < 0.0001; **Figure 5E**]. However, there was no statistically significant correlation between DAO and DAOA protein concentration in the frontal cortex [*r*(49) = 0.27, *p* = 0.06; **Figure 5F**].

### DAO and DAOA Expression across *DAO* and *DAOA* SNP Genotypes in Different Brain Regions

We assessed the potential effect of *DAO* and *DAOA* SNPs on DAO and DAOA expression to understand whether such genetic variations known to be risk factors in schizophrenia play a role in the regulation of their expression. The *DAO* and *DAOA* SNPs were in HWE (*p* > 0.05), and the minor allele frequency (MAF) of *DAO* and *DAOA* SNPs were similar to HapMap CEU MAF (Supplementary Table S2). In the thalamus, *DAO* mRNA was significantly more expressed in rs3918347 *DAO* genotype GG (minor allele G) than in rs3918347 *DAO* genotypes (GA, AA), and *DAO* mRNA was less expressed in rs3918347 *DAO* genotypes GG + GA than in rs3918347 *DAO* genotype AA as assessed by ANCOVA with age as covariate (Supplementary Table S3). However, for the DAO protein, no statistically significant difference was detected among rs3918347 and rs4623951 *DAO* genotypes (Supplementary Table S4). For DAOA protein expression, there was a significantly higher expression in rs3916971 *DAOA* genotype TT (minor allele T) than in rs3916971 *DAOA* genotypes (CT, CC, CT + CC) in the amygdala, as assessed by ANCOVA with age as a covariate (Supplementary Table S5). Nonetheless, from our findings, we cannot conclusively comment on the effect of *DAO* and *DAOA* SNPs on their expression due to their rather small sample size.

### DNA Methylation across *DAO* and *DAOA* CpG Sites in Cerebellum and Frontal Cortex

From our DAO and DAOA mRNA and protein findings, we hypothesized a possible regulation of their expression at the transcriptional levels. To elucidate this, we analyzed *in silico* DNA methylation across *DAO* and *DAOA* CpG sites in the cerebellum and frontal cortex to indirectly explain the differential DAO and DAOA expression in the human brain. In GSE61431 and GSE63347 datasets, gender had no statistically significant effect on DNA methylation levels at *DAO* and *DAOA* CpG sites in the cerebellum and frontal cortex (Mann–Whitney test, *p* > 0.005). In GSE61431 and GSE63347 datasets, *DAO* CpG site cg18037826 is significantly more methylated in the cerebellum than in the frontal cortex as assessed by the Mann–Whitney test, *p* < 0.001 (Supplementary Figure S1A). In the GSE61431 dataset, all studied *DAOA* CpG sites except cg13846327 are statistically significantly (*p* < 0.001) more methylated in the cerebellum than in the frontal cortex (Supplementary Figure S1B). In GSE63347 dataset, *DAOA* CpG sites cg22773522, cg20888753, and cg11374446 are significantly more methylated in the cerebellum than in the frontal cortex (Supplementary Figure S1B). There was no statistically significant correlation between age and DNA methylation levels at *DAO* and *DAOA* CpG sites in the cerebellum and frontal cortex in both studied datasets.

## Discussion

This study investigated the age-span expression of DAO and DAOA mRNA and protein in six brain regions of normal human post-mortem brain samples. In this study, we also determined *in silico* DNA methylation levels at *DAO* and *DAOA* CpG sites in the cerebellum and frontal cortex of control human post-mortem brain. The results of this study have implications for understanding the regulation and expression of *DAO* and *DAOA* genes in the normal human brain during development and aging.

In this study, DAO mRNA and protein was detected in all brain regions studied. *DAO* mRNA was highly expressed in the cerebellum as compared to the other brain regions studied. This finding is in line with previous studies of DAO expression in the human brain (Kapoor et al., 2006; Verrall et al., 2007; GTEx Consortium, 2015). This is the first study which quantified DAO protein in human post-mortem brain using the quantitative ELISA method. Previous studies detected DAO protein in the human brain using methods such as immunohistochemistry and Western blot (Bendikov et al., 2007; Verrall et al., 2007; Uhlen et al., 2015). Immunohistochemical studies measured DAO immunoreactivity in the human brain and found that DAO protein was robustly detected both in the cerebellum and cerebral cortex (Verrall et al., 2007; Uhlen et al., 2015). Another study detected DAO protein in the frontal cortex and hippocampus using Western blot (Bendikov et al., 2007). We found that DAO protein was less expressed in the cerebellum, but *DAO* mRNA was highly expressed in the cerebellum as compared to other regions studied, which might indicate post-transcriptional regulation such as microRNA (miRNA) mediated regulation. The miRWalk 2.0 database (Dweep and Gretz, 2015) with prediction algorithms from four different databases (miRWalk, miRanda, RNA22, Targetscan) showed that there are around 327 predicted miRNA target sites in the promoter region (miRWalk, miRanda, Targetscan), three predicted miRNA target sites in the 5′UTR (miRWalk, miRanda, RNA22), 74 predicted miRNA target sites in the coding DNA sequence (CDS) (miRWalk, miRanda, RNA22, Targetscan), and nine predicted miRNA target sites in the 3′UTR (miRWalk, miRanda, RNA22, Targetscan) of the *DAO* gene. The interaction of these miRNAs with the *DAO* gene in the cerebellum might be the reason for more *DAO* mRNA and less DAO protein. However, these interactions have to be confirmed experimentally. We measured DAO protein but not the activity of DAO protein because the buffer used to isolate proteins deactivated the DAO enzyme. Therefore, in our study, we are unable to comment on the activity of DAO in different brain regions. The DAO protein can be either in an active [flavin adenine dinucleotide (FAD bound) or inactive (FAD unbound) state; Caldinelli et al., 2009; Terry-Lorenzo et al., 2014]. Kapoor et al. (2006) showed that DAO activity was detected in the human cerebellum but not in the human forebrain. Moreover, a recent study by Sasabe et al. (2014) showed that DAO activity was detected in the human hindbrain, midbrain, spinal cord, and only in the white matter of the forebrain. Therefore, DAO activity in the human forebrain is not yet very clear.

*DAOA* is a primate-specific gene which shows a complex alternative splicing pattern, and encodes a protein characterized by a rapidly changing structure during evolution (Chumakov et al., 2002). The DAOA gene and protein expression in the human brain have not yet been fully characterized. We detected DAOA protein using ELISA in all of the brain regions studied. The human protein atlas detected DAOA protein in the cerebellum and cerebral cortex using immunohistochemistry (Uhlen et al., 2015). Kvajo et al. (2008) detected DAOA protein in the human amygdala using Western blot. However, Benzel et al. (2008) could not detect DAOA protein in the human brain (cerebellum, amygdala, frontal cortex) using Western blot. Studies showing *DAOA* mRNA expression in the human brain are limited (Chumakov et al., 2002; Korostishevsky et al., 2006). We were unable to detect *DAOA* mRNA in all the brain regions studied using qRT-PCR, which is in line with previous studies that used methods such as northern blotting, qRT-PCR, and RNA sequencing (Benzel et al., 2008; GTEx Consortium, 2015). The reason for detecting DAOA protein but not *DAOA* mRNA in the human brain might be a tightly regulated DAOA expression or extremely localized expression or post-transcriptional regulation by RNA methylation. A previous study suggested that undetectable *DAOA* mRNA in the human brain might be because of RNA instability motif found in the 5′UTR of *DAOA* gene (Benzel et al., 2008). There are around 314 predicted miRNA target sites in the promoter region (miRWalk, miRanda, Targetscan), five predicted miRNA target sites in the 5′UTR (miRWalk, miRanda, RNA22), 317 predicted miRNA target sites in the CDS (miRWalk, miRanda, Targetscan), and 70 predicted miRNA target sites in the 3′UTR (miRWalk, miRanda, RNA22) of the *DAOA* gene as described in the miRWalk 2.0 database (Dweep and Gretz, 2015) with prediction algorithms from four different databases (miRWalk, miRanda, RNA22, Targetscan). The interaction of these miRNAs with the *DAOA* gene might be the reason for the variation of DAOA protein expression in different brain regions. However, the interactions of miRNA with the *DAOA* gene have to be confirmed experimentally.

During brain development, there are critical and sensitive periods during which the brain is more vulnerable to environmental insults that can potentially lead to psychiatric disorders (Meredith, 2015). In order to understand pathological changes that occur in psychiatric disorders, it is important to analyze the expression of risk genes for psychiatric disorders in the normal brain across the lifespan. In our study, we found that *DAO* mRNA levels were positively correlated with age less than 2 years in the cerebellum and amygdala. Our data is in agreement with previous studies of whole-genome expression analysis in the developing brain which reported that prenatal and neonatal periods are associated with a rapid change of gene expression patterns in the brain as compared to other age periods (Kang et al., 2011; Naumova et al., 2013). During brain development and maturation, synaptic plasticity varies significantly across different brain regions, which might contribute to the brain region-specific gene expression (Huttenlocher and Dabholkar, 1997; Caruana et al., 2012). In this study, DAO expression was brain region specific, indicating that DAO is differentially regulated across hindbrain and forebrain regions. Interestingly, we found a significant positive correlation between DAO and DAOA protein in all the brain regions studied except for the frontal cortex. This positive correlation between DAO and DAOA protein might be because of the variability in density of particular cell types in different brain regions. A recent *in vitro* study showed that there is an interaction between DAO and DAOA proteins (Birolo et al., 2016). Nonetheless, the effect of DAOA protein on DAO is controversial. Chumakov et al. (2002) reported DAOA to be an activator of DAO, but Sacchi et al. (2008) reported DAOA to be a repressor of DAO. From our finding, we are still unable to conclude regarding DAO and DAOA interaction, but we could show their simultaneous expression.

DNA methylation has been widely acknowledged to be crucial for normal brain development (Fan et al., 2001), and alterations in DNA methylome in the human brain might contribute to neuropsychiatric disorders (Illingworth et al., 2015). DNA methylation can lead to both increases and decreases in gene expression (Wagner et al., 2014). We found *in silico* that the cerebellum was significantly more methylated than the frontal cortex in most of the *DAO* and *DAOA* CpG sites which is in line with previous studies (Davies et al., 2012; Hannon et al., 2015; Illingworth et al., 2015). This finding might explain the differential expression of *DAO* mRNA in the cerebellum and frontal cortex. However, further studies are required to understand the extent to which DNA methylation or other epigenetic modifications affect the expression of genes important for human brain function.

In this study, we attempted to carefully address confounding factors such as PMI and gender that are inherent in human post-mortem studies. Our study is the first study which measured concomitantly DAO and DAOA protein levels in human post-mortem brain using quantitative ELISA method. Nevertheless, we were only able to measure total DAO protein but not the DAO activity in different brain regions because of the isolation procedure used. In our study, we had no post-mortem samples between ages 2 and 20 years, therefore we are unable to comment on alterations within this particular developmental phase. We also do not have schizophrenia brain samples to comment on regulation and expression of DAO and DAOA mRNA and protein across different brain regions in schizophrenia. *DAO* and *DAOA* are susceptibility genes for schizophrenia and bipolar disorders. Both disorders are discussed as neurodevelopmental disorders, and first symptoms often occur before early adulthood, whereas the definitive onset of the disorder is typically in early adulthood (Cacabelos and Martinez-Bouza, 2011; Rapoport et al., 2012). Therefore, it is important to investigate the regulation of these genes between 2 and 20 years in the normal human brain. Hence, future studies in this age period would be useful to elucidate this question. Another limitation of the study is that because of limited sample size, it is not powered appropriately, particularly regarding the effect of *DAO* and *DAOA* polymorphisms on their expression. However, the strength of this study lies in the fact that we extracted DNA, RNA, and total protein from the same tissue, which allowed us to investigate concomitantly DAO and DAOA expression in six different brain regions and across a wide range of ages.

In summary, our results showed the regional expression of DAO and DAOA with age in normal human post-mortem brain samples. We detected DAOA protein, despite undetectable *DAOA* mRNA levels in the human post-mortem brain.

## Author Contributions

ZM, C-MM, and EG designed the experiments. VJ performed experiments, analyzed data, and wrote the manuscript. ZM, C-MM, SW, and EG reviewed and revised the manuscript. All authors have approved the final manuscript.

## Conflict of Interest Statement

The authors declare that the research was conducted in the absence of any commercial or financial relationships that could be construed as a potential conflict of interest.

## Footnotes

1 http://www.ncbi.nlm.nih.gov/geo/

## References

[B1] Allen N. C. Bagade S. McQueen M. B. Ioannidis J. P. Kavvoura F. K. Khoury M. J. (2008). Systematic meta-analyses and field synopsis of genetic association studies in schizophrenia: the SzGene database. *Nat. Genet.* 40 827–834. 10.1038/ng.171 18583979

[B2] Almond S. L. Fradley R. L. Armstrong E. J. Heavens R. B. Rutter A. R. Newman R. J. (2006). Behavioral and biochemical characterization of a mutant mouse strain lacking D-amino acid oxidase activity and its implications for schizophrenia. *Mol. Cell. Neurosci.* 32 324–334. 10.1016/j.mcn.2006.05.003 16843004

[B3] Bendikov I. Nadri C. Amar S. Panizzutti R. De Miranda J. Wolosker H. (2007). A CSF and postmortem brain study of D-serine metabolic parameters in schizophrenia. *Schizophr. Res.* 90 41–51. 10.1016/j.schres.2006.10.010 17156977

[B4] Benzel I. Kew J. N. Viknaraja R. Kelly F. de Belleroche J. Hirsch S. (2008). Investigation of G72 (DAOA) expression in the human brain. *BMC Psychiatry* 8:94 10.1186/1471-244X-8-94 PMC2630984 19077230

[B5] Birolo L. Sacchi S. Smaldone G. Molla G. Leo G. Caldinelli L. (2016). Regulating levels of the neuromodulator d-serine in human brain: structural insight into pLG72 and d-amino acid oxidase interaction. *FEBS J.* 283 3353–3370. 10.1111/febs.13809 27400736

[B6] Bradford M. M. (1976). A rapid and sensitive method for the quantitation of microgram quantities of protein utilizing the principle of protein-dye binding. *Anal. Biochem.* 72 248–254. 10.1016/0003-2697(76)90527-3 942051

[B7] Cacabelos R. Martinez-Bouza R. (2011). Genomics and pharmacogenomics of schizophrenia. *CNS Neurosci. Ther.* 17 541–565. 10.1111/j.1755-5949.2010.00187.x 20718829 PMC6493868

[B8] Caldinelli L. Molla G. Sacchi S. Pilone M. S. Pollegioni L. (2009). Relevance of weak flavin binding in human D-amino acid oxidase. *Protein Sci.* 18 801–810. 10.1002/pro.86 19309736 PMC2762592

[B9] Caruana D. A. Alexander G. M. Dudek S. M. (2012). New insights into the regulation of synaptic plasticity from an unexpected place: hippocampal area CA2. *Learn. Mem.* 19 391–400. 10.1101/lm.025304.111 22904370 PMC3418763

[B10] Chang S. L. Hsieh C. H. Chen Y. J. Wang C. M. Shih C. S. Huang P. W. (2014). The C-terminal region of G72 increases D-amino acid oxidase activity. *Int. J. Mol. Sci.* 15 29–43. 10.3390/ijms15010029 PMC3907796 24362575

[B11] Cheng L. Hattori E. Nakajima A. Woehrle N. S. Opal M. D. Zhang C. (2014). Expression of the G72/G30 gene in transgenic mice induces behavioral changes. *Mol. Psychiatry* 19 175–183. 10.1038/mp.2012.185 23337943 PMC3636154

[B12] Chumakov I. Blumenfeld M. Guerassimenko O. Cavarec L. Palicio M. Abderrahim H. (2002). Genetic and physiological data implicating the new human gene G72 and the gene for D-amino acid oxidase in schizophrenia. *Proc. Natl. Acad. Sci. U.S.A.* 99 13675–13680. 10.1073/pnas.182412499 12364586 PMC129739

[B13] Corvin A. McGhee K. A. Murphy K. Donohoe G. Nangle J. M. Schwaiger S. (2007). Evidence for association and epistasis at the DAOA/G30 and D-amino acid oxidase loci in an Irish schizophrenia sample. *Am. J. Med. Genet. B Neuropsychiatr. Genet.* 144B, 949–953. 10.1002/ajmg.b.30452 17492767

[B14] Darby M. M. Yolken R. H. Sabunciyan S. (2016). Consistently altered expression of gene sets in postmortem brains of individuals with major psychiatric disorders. *Transl. Psychiatry* 6 e890. 10.1038/tp.2016.173 PMC5048210 27622934

[B15] Davies M. N. Volta M. Pidsley R. Lunnon K. Dixit A. Lovestone S. (2012). Functional annotation of the human brain methylome identifies tissue-specific epigenetic variation across brain and blood. *Genome Biol.* 13:R43 10.1186/gb-2012-13-6-r43 PMC3446315 22703893

[B16] Detera-Wadleigh S. D. McMahon F. J. (2006). G72/G30 in schizophrenia and bipolar disorder: review and meta-analysis. *Biol. Psychiatry* 60 106–114. 10.1016/j.biopsych.2006.01.019 16581030

[B17] Durrenberger P. F. Fernando F. S. Magliozzi R. Kashefi S. N. Bonnert T. P. Ferrer I. (2012). Selection of novel reference genes for use in the human central nervous system: a BrainNet Europe Study. *Acta Neuropathol.* 124 893–903. 10.1007/s00401-012-1027-z 22864814

[B18] Dweep H. Gretz N. (2015). miRWalk2.0: a comprehensive atlas of microRNA-target interactions. *Nat. Methods* 12 697 10.1038/nmeth.3485 26226356

[B19] Fan G. Beard C. Chen R. Z. Csankovszki G. Sun Y. Siniaia M. (2001). DNA hypomethylation perturbs the function and survival of CNS neurons in postnatal animals. *J. Neurosci.* 21 788–797. 11157065 10.1523/JNEUROSCI.21-03-00788.2001 PMC6762314

[B20] Faul F. Erdfelder E. Buchner A. Lang A. G. (2009). Statistical power analyses using G*Power 3.1: tests for correlation and regression analyses. *Behav. Res. Methods* 41 1149–1160. 10.3758/BRM.41.4.1149 19897823

[B21] Gatt J. M. Burton K. L. Williams L. M. Schofield P. R. (2015). Specific and common genes implicated across major mental disorders: a review of meta-analysis studies. *J. Psychiatr. Res.* 60 1–13. 10.1016/j.jpsychires.2014.09.014 25287955

[B22] Graff J. Kim D. Dobbin M. M. Tsai L. H. (2011). Epigenetic regulation of gene expression in physiological and pathological brain processes. *Physiol. Rev.* 91 603–649. 10.1152/physrev.00012.2010 21527733

[B23] GTEx Consortium (2015). Human genomics. The Genotype-Tissue Expression (GTEx) pilot analysis: multitissue gene regulation in humans. *Science* 348 648–660. 10.1126/science.1262110 25954001 PMC4547484

[B24] Habl G. Zink M. Petroianu G. Bauer M. Schneider-Axmann T. von Wilmsdorff M. (2009). Increased D-amino acid oxidase expression in the bilateral hippocampal CA4 of schizophrenic patients: a post-mortem study. *J. Neural Transm. (Vienna)* 116 1657–1665. 10.1007/s00702-009-0312-z 19823762 PMC2776935

[B25] Hannon E. Lunnon K. Schalkwyk L. Mill J. (2015). Interindividual methylomic variation across blood, cortex, and cerebellum: implications for epigenetic studies of neurological and neuropsychiatric phenotypes. *Epigenetics* 10 1024–1032. 10.1080/15592294.2015.1100786 26457534 PMC4844197

[B26] Hellemans J. Mortier G. De Paepe A. Speleman F. Vandesompele J. (2007). qBase relative quantification framework and software for management and automated analysis of real-time quantitative PCR data. *Genome Biol.* 8:R19 10.1186/gb-2007-8-2-r19 PMC1852402 17291332

[B27] Horvath S. Garagnani P. Bacalini M. G. Pirazzini C. Salvioli S. Gentilini D. (2015). Accelerated epigenetic aging in Down syndrome. *Aging Cell* 14 491–495. 10.1111/acel.12325 25678027 PMC4406678

[B28] Huttenlocher P. R. Dabholkar A. S. (1997). Regional differences in synaptogenesis in human cerebral cortex. *J. Comp. Neurol.* 387 167–178. 10.1002/(SICI)1096-9861(19971020)387:2<167::AID-CNE1>3.0.CO;2-Z 9336221

[B29] Illingworth R. S. Gruenewald-Schneider U. De Sousa D. Webb S. Merusi C. Kerr A. R. (2015). Inter-individual variability contrasts with regional homogeneity in the human brain DNA methylome. *Nucleic Acids Res.* 43 732–744. 10.1093/nar/gku1305 25572316 PMC4333374

[B30] Kang H. J. Kawasawa Y. I. Cheng F. Zhu Y. Xu X. Li M. (2011). Spatio-temporal transcriptome of the human brain. *Nature* 478 483–489. 10.1038/nature10523 22031440 PMC3566780

[B31] Kantrowitz J. T. Javitt D. C. (2010). N-methyl-d-aspartate (n.d.) receptor dysfunction or dysregulation: the final common pathway on the road to schizophrenia? *Brain Res. Bull.* 83 108–121. 10.1016/j.brainresbull.2010.04.006 20417696 PMC2941541

[B32] Kapoor R. Lim K. S. Cheng A. Garrick T. Kapoor V. (2006). Preliminary evidence for a link between schizophrenia and NMDA-glycine site receptor ligand metabolic enzymes, d-amino acid oxidase (DAAO) and kynurenine aminotransferase-1 (KAT-1). *Brain Res.* 1106 205–210. 10.1016/j.brainres.2006.05.082 16828464

[B33] Kleinman J. E. Law A. J. Lipska B. K. Hyde T. M. Ellis J. K. Harrison P. J. (2011). Genetic neuropathology of schizophrenia: new approaches to an old question and new uses for postmortem human brains. *Biol. Psychiatry* 69 140–145. 10.1016/j.biopsych.2010.10.032 21183009 PMC4351748

[B34] Korostishevsky M. Kremer I. Kaganovich M. Cholostoy A. Murad I. Muhaheed M. (2006). Transmission disequilibrium and haplotype analyses of the G72/G30 locus: suggestive linkage to schizophrenia in Palestinian Arabs living in the North of Israel. *Am. J. Med. Genet. B Neuropsychiatr. Genet.* 141B, 91–95. 10.1002/ajmg.b.30212 16082701

[B35] Kvajo M. Dhilla A. Swor D. E. Karayiorgou M. Gogos J. A. (2008). Evidence implicating the candidate schizophrenia/bipolar disorder susceptibility gene G72 in mitochondrial function. *Mol. Psychiatry* 13 685–696. 10.1038/sj.mp.4002052 17684499

[B36] Labrie V. Roder J. C. (2010). The involvement of the NMDA receptor D-serine/glycine site in the pathophysiology and treatment of schizophrenia. *Neurosci. Biobehav. Rev.* 34 351–372. 10.1016/j.neubiorev.2009.08.002 19695284

[B37] Labrie V. Wong A. H. Roder J. C. (2012). Contributions of the D-serine pathway to schizophrenia. *Neuropharmacology* 62 1484–1503. 10.1016/j.neuropharm.2011.01.030 21295046

[B38] Liu Y. L. Wang S. C. Hwu H. G. Fann C. S. Yang U. C. Yang W. C. (2016). Haplotypes of the D-amino acid oxidase gene are significantly associated with schizophrenia and its neurocognitive deficits. *PLoS ONE* 11:e0150435 10.1371/journal.pone.0150435 PMC4795637 26986737

[B39] Matsui T. Sekiguchi M. Hashimoto A. Tomita U. Nishikawa T. Wada K. (1995). Functional comparison of D-serine and glycine in rodents: the effect on cloned NMDA receptors and the extracellular concentration. *J. Neurochem.* 65 454–458. 10.1046/j.1471-4159.1995.65010454.x 7790891

[B40] Meredith R. M. (2015). Sensitive and critical periods during neurotypical and aberrant neurodevelopment: a framework for neurodevelopmental disorders. *Neurosci. Biobehav. Rev.* 50 180–188. 10.1016/j.neubiorev.2014.12.001 25496903

[B41] Miller A. Shen J. Masse L. C. (2016). Child functional characteristics explain child and family outcomes better than diagnosis: population-based study of children with autism or other neurodevelopmental disorders/disabilities. *Health Rep.* 27 9–18. 27305076

[B42] Mossner R. Schuhmacher A. Wagner M. Quednow B. B. Frommann I. Kuhn K. U. (2010). DAOA/G72 predicts the progression of prodromal syndromes to first episode psychosis. *Eur. Arch. Psychiatry Clin. Neurosci.* 260 209–215. 10.1007/s00406-009-0044-y 19763662 PMC3128744

[B43] Naumova O. Y. Lee M. Rychkov S. Y. Vlasova N. V. Grigorenko E. L. (2013). Gene expression in the human brain: the current state of the study of specificity and spatiotemporal dynamics. *Child Dev.* 84 76–88. 10.1111/cdev.12014 23145569 PMC3557706

[B44] Numata S. Ye T. Hyde T. M. Guitart-Navarro X. Tao R. Wininger M. (2012). DNA methylation signatures in development and aging of the human prefrontal cortex. *Am. J. Hum. Genet.* 90 260–272. 10.1016/j.ajhg.2011.12.020 22305529 PMC3276664

[B45] Ono K. Shishido Y. Park H. K. Kawazoe T. Iwana S. Chung S. P. (2009). Potential pathophysiological role of D-amino acid oxidase in schizophrenia: immunohistochemical and in situ hybridization study of the expression in human and rat brain. *J Neural Transm (Vienna)* 116 1335–1347. 10.1007/s00702-009-0289-7 19685198

[B46] Otte D. M. Rasko T. Wang M. Dreiseidler M. Drews E. Schrage H. (2014). Identification of the mitochondrial MSRB2 as a binding partner of LG72. *Cell Mol. Neurobiol.* 34 1123–1130. 10.1007/s10571-014-0087-0 25078755 PMC11488933

[B47] Pidsley R. Viana J. Hannon E. Spiers H. Troakes C. Al-Saraj S. (2014). Methylomic profiling of human brain tissue supports a neurodevelopmental origin for schizophrenia. *Genome Biol.* 15 483 10.1186/s13059-014-0483-2 PMC4262979 25347937

[B48] Pollegioni L. Piubelli L. Sacchi S. Pilone M. S. Molla G. (2007). Physiological functions of D-amino acid oxidases: from yeast to humans. *Cell Mol. Life. Sci.* 64 1373–1394. 10.1007/s00018-007-6558-4 17396222 PMC11136250

[B49] Prata D. Breen G. Osborne S. Munro J. St Clair D. Collier D. (2008). Association of DAO and G72(DAOA)/G30 genes with bipolar affective disorder. *Am. J. Med. Genet. B Neuropsychiatr..* 147B, 914–917. 10.1002/ajmg.b.30682 18165970

[B50] Purcell S. Neale B. Todd-Brown K. Thomas L. Ferreira M. A. Bender D. (2007). PLINK: a tool set for whole-genome association and population-based linkage analyses. *Am. J. Hum. Genet.* 81 559–575. 10.1086/519795 17701901 PMC1950838

[B51] Rapoport J. L. Giedd J. N. Gogtay N. (2012). Neurodevelopmental model of schizophrenia: update 2012. *Mol. Psychiatry* 17 1228–1238. 10.1038/mp.2012.23 22488257 PMC3504171

[B52] Ruijter J. M. Ramakers C. Hoogaars W. M. Karlen Y. Bakker O. van den Hoff M. J. (2009). Amplification efficiency: linking baseline and bias in the analysis of quantitative PCR data. *Nucleic Acids Res.* 37:e45 10.1093/nar/gkp045 PMC2665230 19237396

[B53] Sacchi S. Bernasconi M. Martineau M. Mothet J. P. Ruzzene M. Pilone M. S. (2008). pLG72 modulates intracellular D-serine levels through its interaction with D-amino acid oxidase: effect on schizophrenia susceptibility. *J. Biol. Chem.* 283 22244–22256. 10.1074/jbc.M709153200 18544534

[B54] Sacchi S. Caldinelli L. Cappelletti P. Pollegioni L. Molla G. (2012). Structure-function relationships in human D-amino acid oxidase. *Amino Acids* 43 1833–1850. 10.1007/s00726-012-1345-4 22865246

[B55] Sacchi S. Cappelletti P. Giovannardi S. Pollegioni L. (2011). Evidence for the interaction of D-amino acid oxidase with pLG72 in a glial cell line. *Mol. Cell. Neurosci.* 48 20–28. 10.1016/j.mcn.2011.06.001 21679769

[B56] Sasabe J. Suzuki M. Imanishi N. Aiso S. (2014). Activity of D-amino acid oxidase is widespread in the human central nervous system. *Front. Synaptic Neurosci.* 6:14 10.3389/fnsyn.2014.00014 PMC4050652 24959138

[B57] Schizophrenia Working Group of the Psychiatric Genomics Consortium (2014). Biological insights from 108 schizophrenia-associated genetic loci. *Nature* 511 421–427. 10.1038/nature13595 25056061 PMC4112379

[B58] Shleper M. Kartvelishvily E. Wolosker H. (2005). D-serine is the dominant endogenous coagonist for NMDA receptor neurotoxicity in organotypic hippocampal slices. *J. Neurosci.* 25 9413–9417. 10.1523/JNEUROSCI.3190-05.2005 16221850 PMC6725696

[B59] Sibille E. (2013). Molecular aging of the brain, neuroplasticity, and vulnerability to depression and other brain-related disorders. *Dialogues Clin. Neurosci.* 15 53–65. 23576889 10.31887/DCNS.2013.15.1/esibille PMC3622469

[B60] Terry-Lorenzo R. T. Chun L. E. Brown S. P. Heffernan M. L. Fang Q. K. Orsini M. A. (2014). Novel human D-amino acid oxidase inhibitors stabilize an active-site lid-open conformation. *Biosci. Rep.* 34 e00133 10.1042/BSR20140071 PMC4127593 25001371

[B61] Uhlen M. Fagerberg L. Hallstrom B. M. Lindskog C. Oksvold P. Mardinoglu A. (2015). Proteomics. Tissue-based map of the human proteome. *Science* 347:1260419 10.1126/science.1260419 25613900

[B62] Verrall L. Burnet P. W. Betts J. F. Harrison P. J. (2010). The neurobiology of D-amino acid oxidase and its involvement in schizophrenia. *Mol. Psychiatry* 15 122–137. 10.1038/mp.2009.99 19786963 PMC2811712

[B63] Verrall L. Walker M. Rawlings N. Benzel I. Kew J. N. Harrison P. J. (2007). d-Amino acid oxidase and serine racemase in human brain: normal distribution and altered expression in schizophrenia. *Eur. J. Neurosci.* 26 1657–1669. 10.1111/j.1460-9568.2007.05769.x 17880399 PMC2121142

[B64] Wagner J. R. Busche S. Ge B. Kwan T. Pastinen T. Blanchette M. (2014). The relationship between DNA methylation, genetic and expression inter-individual variation in untransformed human fibroblasts. *Genome Biol.* 15:R37 10.1186/gb-2014-15-2-r37 PMC4053980 24555846

