# Supplementary material for: Expression of D-Amino Acid Oxidase (DAO/DAAO) and D-Amino Acid Oxidase Activator (DAOA/G72) during Development and Aging in the Human Post-mortem Brain
Source: Front Neuroanat. 2017 Apr 6;11:31. doi: 10.3389/fnana.2017.00031 (PMC5382383; doi:10.3389/fnana.2017.00031)
Supplement: Supplementary file 1 [file Presentation_1.PDF]

Supplementary Material

Expression of D-amino acid oxidase (DAO/DAAO) and D-amino acid oxidase activator (DAOA/G72) during Development and Aging in the Human Post-Mortem Brain

Vinita Jagannath[1], Zoya Marinova[1], Camelia-Maria Monoranu[2], Susanne Walitza[1], Edna Grünblatt[1*]

*** Correspondence:** Edna Grünblatt: edna.gruenblatt@kjpd.uzh.ch

1 Supplementary Methods, Results, Figures and Tables

1.1 Supplementary Methods

S1 Sodium dodecyl sulfate polyacrylamide gel electrophoresis (SDS-PAGE) and Western blot

The acetone precipitated proteins from all six brain regions of subject 081/01 (3 months male) were subjected to electrophoresis on 10–20% Tris-HCl Gel (Bio-Rad) and transferred to PVDF membrane (Bio-Rad). The membrane was blocked with 5% non-fat milk in 1x phosphate buffered saline (PBS) pH 7.4 (Gibco) with 0.1% Tween-20 (Sigma-Aldrich) for 2 hours at room temperature and then it was incubated with primary antibody against DAO (1 µg/ml, same primary antibody that was used in DAO ELISA kit, SEJ298Hu; Cloud-Clone Corp.) or DAOA (1 µg/ml, same primary antibody that was used in DAOA ELISA kit, SEJ297Hu; Cloud-Clone Corp.) at 4°C overnight. The membrane was washed thrice with PBS-T (1x PBS with 0.1% Tween-20) and then incubated with anti-rabbit secondary HRP-conjugated antibody (1:5000, ab97051, abcam) for one hour at room temperature. The protein visualisation was carried out with 20X LumiGLO® Reagent and 20X Peroxide (Cell Signaling) by exposing the membrane to X-ray film in cassette for 1 minute.

1.2 Supplementary Results

To prove the specificity of DAO and DAOA ELISA kits, we performed SDS-PAGE and western blot with acetone precipitated samples of subject 081/01 (3 month old male) from six brain regions. We found that DAO and DAOA proteins were expressed in all six brain regions at the expected sizes of 40 kDa (supplementary figure S2A) and 20 kDa (supplementary figure S2B) respectively. This finding proves the specificity of the primary antibody used in DAO and DAOA ELISA kits.

1.3 Supplementary Figures

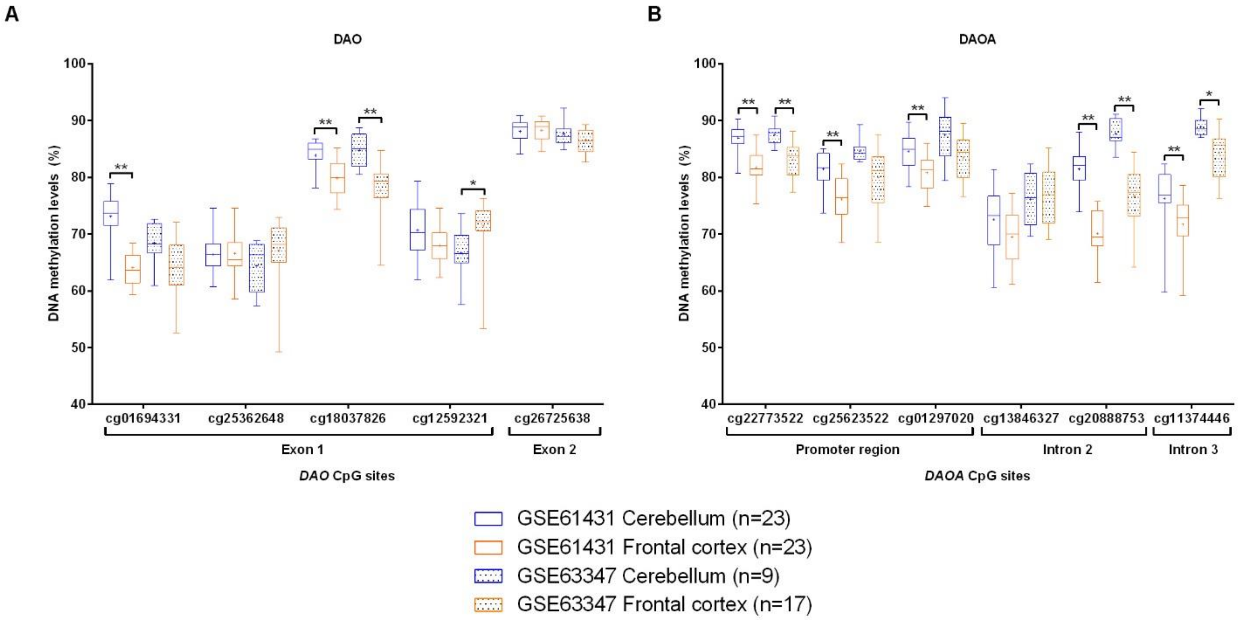

Supplementary Figure S1. Percentage of DNA methylation across *DAO* (A) and *DAOA* (B) CpG sites in cerebellum and frontal cortex of human brain reported in GEO datasets GSE61431 and GSE63347. Values are presented as box and whisker plots, whiskers represent minimum to maximum values, '+' indicates mean values. Differences in DNA methylation levels between cerebellum and frontal cortex at each CpG site was assessed by the Mann-Whitney test (*p<0.005, **p<0.001)

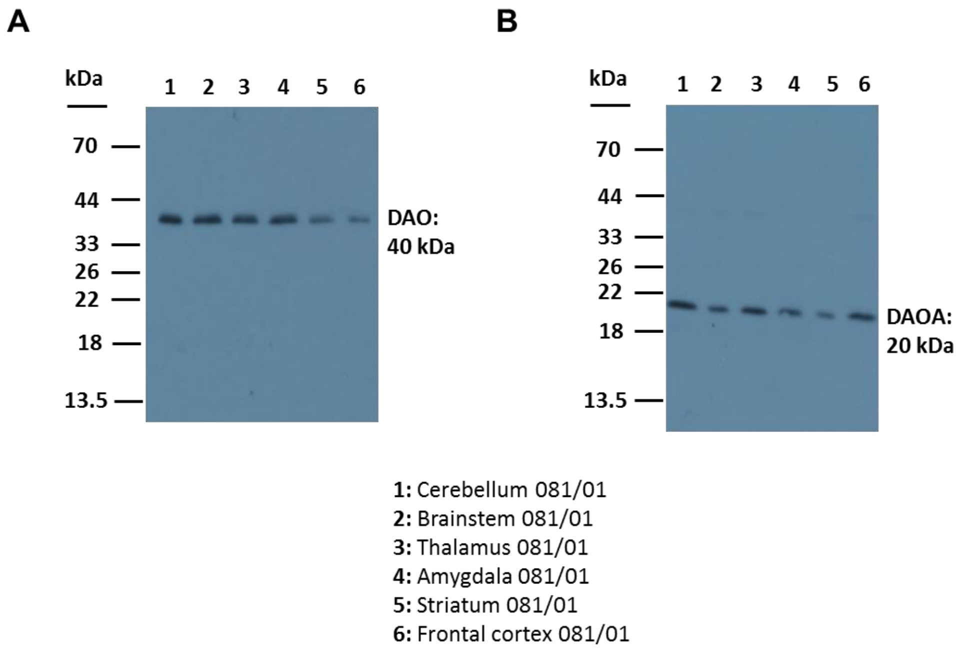

Supplementary Figure S2. Expression of DAO (A) and DAOA (B) proteins across six brain regions of subject 081/01. DAO and DAOA proteins are detected in all studied brain regions at the expected sizes of 40 kDa (A) and 20 kDa (B) respectively.

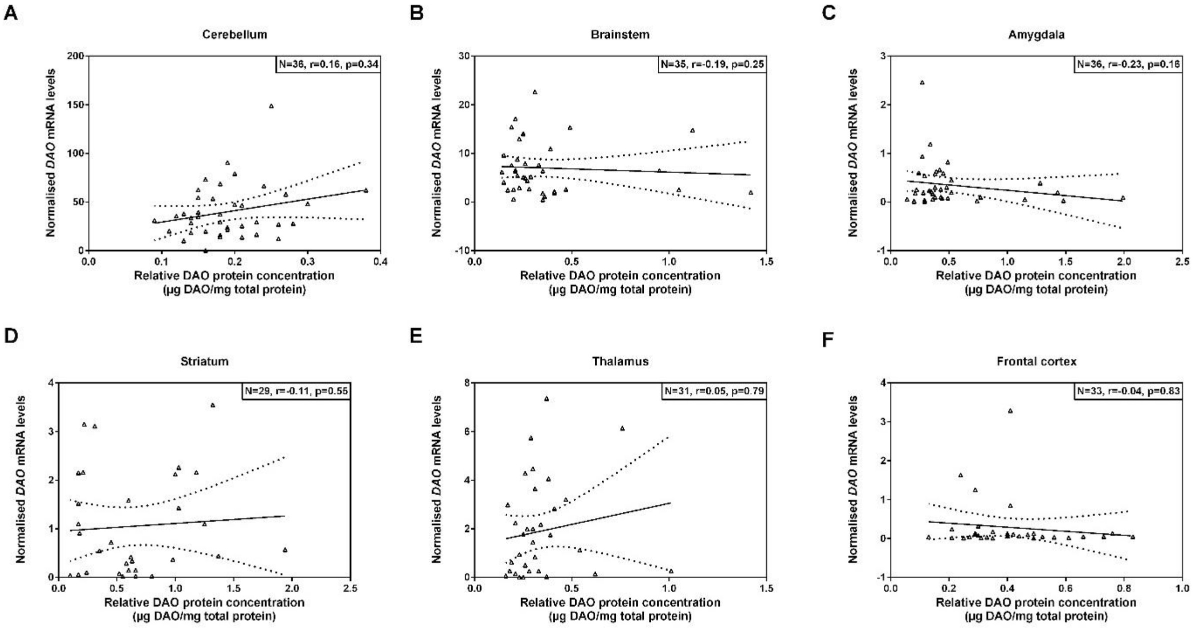

Supplementary Figure S3. Correlation between *DAO* mRNA and DAO protein expression (µg DAO/mg total protein) in six regions of the human post-mortem brain (A-F). Data is presented as scatter plots with linear fit and 95% confidence intervals. Correlation between *DAO* mRNA and DAO protein levels in different brain regions were assessed with the partial correlation test, controlled for age; $p<0.05$ was taken as statistically significant (N: sample size, r: correlation co-efficient)

1.4 Supplementary Tables

Table S1 Demographic data of the study population by age groups

Age groups	N	Gender		Age (mean±SD)	PMI (mean±SD)
		Male N (%)	Female N (%)		
Prenatal	11	4 (36%)	7 (64%)	21.3±6.9 g.w	36.9±19.9 hours
0-2 years	11	6 (55%)	5 (45%)	0.62±0.60 years	25.5±7.7 hours
20-35 years	4	3 (75%)	1 (25%)	30.5±5.8 years	27.3±20.8 hours
36-60 years	12	6 (50%)	6 (50%)	49.8±6.1 years	26.3±12.1 hours
>61 years	17	11 (65%)	6 (35%)	73.6±8.6 years	25.9±10.2 hours

PMI: post-mortem interval; SD: standard deviation; g.w: gestational weeks

Table S2 Genotype distribution and minor allele frequencies of *DAO* and *DAOA* SNPs

Gene	SNP ID	Genotype distribution			Minor allele	MAF	HapMap CEU MAF
		Genotype	N	H-W p-value			
DAO	rs3918347	GG	3	1	G	0.25	0.274
		GA	22				
		AA	31				
	rs4623951	CC	6	0.096	C	0.411	0.412
		CT	34				
		TT	16				
DAOA	rs3916971	TT	13	1	T	0.482	0.394
		CT	28				
		CC	15				
	rs778293	GG	10	0.791	G	0.438	0.389
		AG	29				
		AA	17				
	rs746187	CC	6	0.520	C	0.295	0.375
		CT	21				
		TT	29				

H-W: Hardy-Weinberg; MAF: minor allele frequency; SNP: single nucleotide polymorphism

Table S3 Differences in *DAO* mRNA levels among rs3918347 and rs4623951 *DAO* SNP genotypes across six brain regions of human post-mortem brain

DAO SNP ID	Brain regions	Normalised *DAO* mRNA levels								
		Genotypes (N)	Mean± SEM	p-value[a]	Dom model	Mean± SEM	p-value[a]	Rec model	Mean± SEM	p-value[a]
rs3918347	Cb	GG (2)	34.7±18.6	0.378	GG+GA	37.1±4.84	0.276	GG	34.7±18.6	0.272
		GA (19)	37.4±5.20					GA+AA	39.6±4.16	
		AA (23)	41.4±6.40		AA	41.4±6.40				
	BS	GG (2)	7.11±1.67	0.366	GG+GA	6.06±0.93	0.170	GG	7.11±1.67	0.506
		GA (16)	5.93±1.03					GA+AA	6.90±0.88	
		AA (22)	7.62±1.33		AA	7.62±1.33				
	Amg	GG (2)	0.39±0.23	0.880	GG+GA	0.25±0.06	0.638	GG	0.39±0.23	0.739
		GA (14)	0.23±0.06					GA+AA	0.33±0.08	
		AA (23)	0.40±0.11		AA	0.40±0.11				
	Str	GG (2)	2.63±0.48	0.728	GG+GA	1.02±0.26	0.636	GG	2.63±0.48	0.610
		GA (16)	0.82±0.24					GA+AA	0.89±0.17	
		AA (17)	0.95±0.23		AA	0.95±0.23				
	TH	GG (2)	3.44±0.62	**0.049***	GG+GA	1.32±0.30	**0.017***	GG	3.44±0.62	0.970
		GA (14)	1.02±0.25					GA+AA	1.82±0.33	
		AA (21)	2.36±0.49		AA	2.36±0.49				
	FC	GG (2)	0.06±0.03	0.313	GG+GA	0.14±0.08	0.126	GG	0.06±0.03	0.537
		GA (13)	0.16±0.09					GA+AA	0.27±0.11	
		AA (21)	0.35±0.17		AA	0.35±0.17				
rs4623951	Cb	CC (6)	55.4±20.7	0.623	CC+CT	36.3±4.71	0.578	CC	55.4±20.7	0.527
		CT (28)	32.2±3.5					CT+TT	36.9±3.35	
		TT (10)	49.9±6.76		TT	49.9±6.76				
	BS	CC (4)	8.15±2.44	0.902	CC+CT	6.63±0.93	0.980	CC	8.15±2.44	0.659
		CT (25)	6.39±1.02					CT+TT	6.78±0.90	
		TT (11)	7.66±1.87		TT	7.66±1.87				
	Amg	CC (5)	0.36±0.17	0.960	CC+CT	0.36±0.09	0.776	CC	0.36±0.17	0.969
		CT (24)	0.36±0.11					CT+TT	0.33±0.08	
		TT (10)	0.28±0.07		TT	0.28±0.07				
	Str	CC (5)	1.26±0.62	0.839	CC+CT	0.88±0.20	0.561	CC	1.26±0.62	0.766
		CT (20)	0.79±0.21					CT+TT	0.94±0.18	
		TT (10)	1.26±0.32		TT	1.26±0.32				
	TH	CC (5)	2.51±0.95	0.662	CC+CT	1.98±0.39	0.403	CC	2.51±0.95	0.556
		CT (20)	1.84±0.44					CT+TT	1.82±0.34	
		TT (12)	1.78±0.55		TT	1.78±0.55				
	FC	CC (6)	0.23±0.13	0.771	CC+CT	0.33±0.14	0.553	CC	0.23±0.13	0.572
		CT (21)	0.35±0.17					CT+TT	0.27±0.12	
		TT (9)	0.07±0.02		TT	0.07±0.02				

[a] p-value by ANCOVA with age as a covariate; *p<0.05 (bold font)

SEM: standard error of the mean; Dom model: dominant model; Rec model: recessive model; Cb: cerebellum; BS: brainstem; Amg: amygdala; Str: striatum; TH: thalamus; FC: frontal cortex

Table S4 Differences in DAO protein levels among rs3918347 and rs4623951 *DAO* SNP genotypes across six brain regions of human post-mortem brain

DAO SNP ID	Brain regions	Relative DAO protein concentration (µg DAO/mg total protein)								
		Genotypes (N)	Mean± SEM	p-value[a]	Dom model	Mean± SEM	p-value[a]	Rec model	Mean± SEM	p-value[a]
rs3918347	Cb	GG (2)	0.18±0.01	0.612	GG+GA	0.19±0.01	0.585	GG	0.18±0.01	0.353
		GA (18)	0.19±0.01					GA+AA	0.19±0.01	
		AA (19)	0.19±0.01		AA	0.19±0.01				
	BS	GG (2)	0.22±0.01	0.428	GG+GA	0.34±0.06	0.563	GG	0.22±0.01	0.205
		GA (20)	0.35±0.07					GA+AA	0.38±0.05	
		AA (25)	0.41±0.08		AA	0.41±0.08				
	Amg	GG (3)	0.34±0.04	0.624	GG+GA	0.44±0.06	0.546	GG	0.34±0.04	0.363
		GA (18)	0.45±0.07					GA+AA	0.49±0.06	
		AA (25)	0.51±0.09		AA	0.51±0.09				
	Str	GG (3)	0.30±0.07	0.637	GG+GA	0.49±0.07	0.507	GG	0.30±0.07	0.631
		GA (20)	0.51±0.08					GA+AA	0.54±0.07	
		AA (25)	0.56±0.10		AA	0.56±0.10				
	TH	GG (3)	0.41±0.02	0.911	GG+GA	0.37±0.07	0.680	GG	0.41±0.02	0.804
		GA (20)	0.36±0.08					GA+AA	0.39±0.05	
		AA (25)	0.41±0.06		AA	0.41±0.06				
	FC	GG (3)	0.37±0.11	0.545	GG+GA	0.38±0.04	0.713	GG	0.37±0.11	0.270
		GA (22)	0.38±0.04					GA+AA	0.38±0.02	
		AA (29)	0.37±0.03		AA	0.37±0.03				
rs4623951	Cb	CC (6)	0.21±0.02	0.632	CC+CT	0.19±0.01	0.392	CC	0.21±0.02	0.499
		CT (24)	0.19±0.01					CT+TT	0.19±0.01	
		TT (9)	0.19±0.01		TT	0.19±0.01				
	BS	CC (6)	0.31±0.07	0.707	CC+CT	0.35±0.05	0.697	CC	0.31±0.07	0.547
		CT (28)	0.36±0.06					CT+TT	0.39±0.06	
		TT (13)	0.45±0.14		TT	0.45±0.14				
	Amg	CC (5)	0.34±0.07	0.498	CC+CT	0.47±0.07	0.795	CC	0.34±0.07	0.289
		CT (29)	0.49±0.08					CT+TT	0.49±0.06	
		TT (12)	0.49±0.09		TT	0.49±0.09				
	Str	CC (5)	0.49±0.16	0.723	CC+CT	0.51±0.07	0.954	CC	0.49±0.16	0.442
		CT (31)	0.51±0.08					CT+TT	0.53±0.07	
		TT (12)	0.58±0.15		TT	0.58±0.15				
	TH	CC (6)	0.45±0.08	0.957	CC+CT	0.38±0.05	0.776	CC	0.45±0.08	0.870
		CT (28)	0.37±0.06					CT+TT	0.38±0.05	
		TT (14)	0.40±0.10		TT	0.40±0.10				
	FC	CC (6)	0.36±0.06	0.892	CC+CT	0.35±0.02	0.895	CC	0.36±0.06	0.679
		CT (33)	0.35±0.03					CT+TT	0.38±0.03	
		TT (15)	0.44±0.06		TT	0.44±0.06				

[a] p-value by ANCOVA with age as a covariate; *p<0.05 (bold font)

SEM: standard error of the mean; Dom model: dominant model; Rec model: recessive model; Cb: cerebellum; BS: brainstem; Amg: amygdala; Str: striatum; TH: thalamus; FC: frontal cortex

Table S5 Differences in DAOA protein levels among rs3916971, rs778293 and rs746187 *DAOA* SNP genotypes across six brain regions of human post-mortem brain

DAOA SNP ID	Brain regions	Relative DAOA protein concentration (µg DAOA/mg total protein)								
		Genotypes (N)	Mean± SEM	p-value[a]	Dom model	Mean± SEM	p-value[a]	Rec model	Mean± SEM	p-value[a]
rs3916971	Cb	TT (6)	0.02±0.002	0.776	TT+CT	0.02±0.002	0.791	TT	0.02±0.002	0.475
		CT (21)	0.02±0.003					CT+CC	0.02±0.002	
		CC (13)	0.02±0.002		CC	0.02±0.002				
	BS	TT (9)	0.05±0.018	0.623	TT+CT	0.03±0.006	0.893	TT	0.05±0.018	0.383
		CT (23)	0.03±0.003					CT+CC	0.03±0.003	
		CC (14)	0.03±0.007		CC	0.03±0.007				
	Amg	TT (7)	0.05±0.021	**0.017***	TT+CT	0.03±0.007	0.719	TT	0.05±0.021	**0.012***
		CT (18)	0.02±0.002					CT+CC	0.02±0.002	
		CC (14)	0.02±0.003		CC	0.02±0.003				
	Str	TT (10)	0.03±0.009	0.922	TT+CT	0.04±0.005	0.935	TT	0.03±0.009	0.686
		CT (26)	0.04±0.006					CT+CC	0.04±0.005	
		CC (12)	0.04±0.009		CC	0.04±0.009				
	TH	TT (10)	0.02±0.004	0.657	TT+CT	0.02±0.003	0.848	TT	0.02±0.004	0.432
		CT (23)	0.03±0.005					CT+CC	0.02±0.003	
		CC (14)	0.02±0.001		CC	0.02±0.001				
	FC	TT (13)	0.03±0.005	0.603	TT+CT	0.03±0.002	0.339	TT	0.03±0.005	0.528
		CT (26)	0.03±0.003					CT+CC	0.03±0.003	
		CC (13)	0.04±0.006		CC	0.04±0.006				
rs778293	Cb	GG (5)	0.02±0.002	0.415	GG+AG	0.02±0.002	0.221	GG	0.02±0.002	0.937
		AG (21)	0.02±0.003					AG+AA	0.02±0.002	
		AA (14)	0.02±0.002		AA	0.02±0.002				
	BS	GG (7)	0.05±0.019	0.241	GG+AG	0.03±0.006	0.123	GG	0.05±0.019	0.237
		AG (23)	0.03±0.005					AG+AA	0.03±0.004	
		AA (16)	0.03±0.007		AA	0.03±0.007				
	Amg	GG (6)	0.04±0.017	0.300	GG+AG	0.03±0.007	0.143	GG	0.04±0.017	0.332
		AG (20)	0.03±0.007					AG+AA	0.02±0.004	
		AA (13)	0.02±0.001		AA	0.02±0.001				
	Str	GG (8)	0.04±0.009	0.538	GG+AG	0.04±0.005	0.637	GG	0.04±0.009	0.400
		AG (26)	0.04±0.006					AG+AA	0.04±0.005	
		AA (14)	0.04±0.008		AA	0.04±0.008				
	TH	GG (9)	0.02±0.004	0.549	GG+AG	0.02±0.003	0.390	GG	0.02±0.004	0.756
		AG (22)	0.02±0.004					AG+AA	0.02±0.003	
		AA (16)	0.02±0.004		AA	0.02±0.004				
	FC	GG (10)	0.03±0.007	0.689	GG+AG	0.03±0.003	0.670	GG	0.03±0.007	0.556
		AG (27)	0.03±0.003					AG+AA	0.03±0.002	
		AA (15)	0.03±0.004		AA	0.03±0.004				
rs746187	Cb	CC (4)	0.03±0.011	0.149	CC+CT	0.02±0.003	0.648	CC	0.03±0.011	0.051
		CT (15)	0.02±0.002					CT+TT	0.02±0.001	
		TT (21)	0.02±0.001		TT	0.02±0.001				
	BS	CC (5)	0.04±0.011	0.679	CC+CT	0.03±0.006	0.764	CC	0.04±0.011	0.376
		CT (18)	0.03±0.006					CT+TT	0.03±0.005	
		TT (23)	0.03±0.007		TT	0.03±0.007				
	Amg	CC (3)	0.01±0.003	0.193	CC+CT	0.03±0.006	0.340	CC	0.01±0.003	0.256
		CT (14)	0.03±0.008					CT+TT	0.03±0.005	
		TT (22)	0.02±0.006		TT	0.02±0.006				
	Str	CC (6)	0.05±0.017	0.631	CC+CT	0.04±0.007	0.354	CC	0.05±0.017	0.546
		CT (18)	0.04±0.007					CT+TT	0.04±0.004	
		TT (24)	0.03±0.005		TT	0.03±0.005				
	TH	CC (5)	0.04±0.012	0.164	CC+CT	0.03±0.004	0.366	CC	0.04±0.012	0.058
		CT (18)	0.02±0.005					CT+TT	0.02±0.002	
		TT (24)	0.02±0.002		TT	0.02±0.002				
	FC	CC (6)	0.03±0.004	0.572	CC+CT	0.03±0.003	0.388	CC	0.03±0.004	0.788
		CT (21)	0.03±0.004					CT+TT	0.03±0.003	
		TT (25)	0.03±0.004		TT	0.03±0.004				

[a] p-value by ANCOVA with age as a covariate; *p<0.05 (bold font); Dom model: dominant model; Rec model: recessive model; Cb: cerebellum; BS: brainstem; Amg: amygdala; Str: striatum; TH: thalamus; FC: frontal cortex

Table S6 Detailed demographic characteristics of the study population

Sl No.	Gender	Age	PMI (hours)	Cause of death	Brain bank
1	Female	59 yr	24	Pneumonia, cardiac failure	Wurzburg
2	Male	66 yr	19	Cardiovascular failure	Wurzburg
3	Male	65 yr	25	Respiratory insufficiency	Wurzburg
4	Female	0.42 yr	24	Cardiac arrest	Wurzburg
5	Female	84 yr	32	Cardiovascular failure, shock	Wurzburg
6	Male	35 yr	58	Sudden cardiac death	Wurzburg
7	Male	49 yr	14	Haemorrhagic shock, spleen rupture	Wurzburg
8	Female	60 yr	9	Toxic multi-organ failure	Wurzburg
9	Male	73 yr	24	Cardiovascular failure, sepsis	Wurzburg
10	Female	45 yr	41	Sudden cardiac death	Wurzburg
11	Female	41 g.w	7	Aspiration of amniotic fluid, respiratory insufficiency	Wurzburg
12	Female	64 yr	32	Cardiovascular failure, septic shock	Wurzburg
13	Male	0.25 yr	24	Cardiovascular failure	Wurzburg
14	Female	38 yr	20	Aneurism rupture	Wurzburg
15	Male	63 yr	24	Bleeding oesophageal varices	Wurzburg
16	Female	91 yr	12	Circulatory failure	Wurzburg
17	Male	66 yr	23	Acute cardiovascular failure	Wurzburg
18	Female	73 yr	24	Acute myocardial infarction	Wurzburg
19	Female	76 yr	20	Cardiovascular failure	Wurzburg
20	Female	51 yr	12.5	Multi-organ failure	Wurzburg
21	Male	84 yr	48	Acute myocardial infarction	Wurzburg
22	Female	33 yr	18	Multi-organ failure	Wurzburg
23	Male	0.42 yr	48	Respiratory insufficiency	Wurzburg
24	Male	85 yr	20.5	Cardiac arrest	Wurzburg
25	Female	0.125 yr	24	Acute cardiovascular failure	Wurzburg
26	Male	22 yr	21	Cardiovascular failure, sepsis	Wurzburg
27	Female	73 yr	24	Multi-organ failure	Wurzburg
28	Male	46 yr	19	Cardiovascular failure	Wurzburg
29	Male	80 yr	23	Cardiac failure	Wurzburg
30	Male	51 yr	42	Cardiovascular failure	Wurzburg
31	Male	54 yr	27	Respiratory insufficiency	Wurzburg
32	Male	52 yr	44	Myocardial infarction	Wurzburg
33	Male	64 yr	50	Respiratory insufficiency	Wurzburg
34	Male	32 yr	12	Cardiac failure	Wurzburg
35	Male	0.75 yr	24	Cardiovascular failure, septic shock	Wurzburg
36	Male	74 yr	12	Circulatory failure	Wurzburg
37	Male	71 yr	27	Pulmonary embolism	Wurzburg
38	Female	46 yr	27	Cardiac failure	Wurzburg
39	Male	47 yr	36	Cardiac failure after acute myocardial infarction	Wurzburg
40	Female	0.25 yr	17	Accidental asphyxia	London
41	Male	0.375 yr	24	Coronary heart disease	London
42	Male	1.83 yr	24	Coronary heart disease	London
43	Female	1.75 yr	24	Coronary heart disease	London
44	Female	0.29 yr	24	Coronary heart disease	London
45	Male	0.33 yr	24	Coronary heart disease	London
46	Female	20 g.w	36	Spontaneous abortion	London
47	Male	17 g.w	62	Spontaneous abortion	London
48	Male	18 g.w	24	Spontaneous abortion	London
49	Female	18 g.w	14	Unknown	London
50	Female	20 g.w	65	Prostaglandin induction/polydactyly	London
51	Male	19 g.w	65	Unknown	London
52	Female	22 g.w	32	Spontaneous abortion	London
53	Female	23 g.w	42	Unknown	London
54	Male	20 g.w	32	Spontaneous miscarriage	London
55	Female	16 g.w	27	Unknown	London

PMI: post-mortem interval; yr: years; g.w: gestational weeks

Table S7 Power analysis for *DAO* mRNA, DAO and DAOA protein expression across age groups

Brain regions	Age groups	***DAO* mRNA**		**DAO protein**		**DAOA protein**	
		**Effect size**	**Power**	**Effect size**	**Power**	**Effect size**	**Power**
Cerebellum	Prenatal	0.53	0.77	0.55	0.73	0.35	0.34
	0-2 yr						
	20-35 yr						
	36-60 yr						
	>61 yr						
Brainstem	Prenatal	0.42	0.49	0.30	0.30	0.41	0.54
	0-2 yr						
	20-35 yr						
	36-60 yr						
	>61 yr						
Amygdala	Prenatal	0.31	0.26	0.30	0.30	0.31	0.26
	0-2 yr						
	20-35 yr						
	36-60 yr						
	>61 yr						
Striatum	Prenatal	0.57	0.78	0.45	0.65	0.35	0.43
	0-2 yr						
	20-35 yr						
	36-60 yr						
	>61 yr						
Thalamus	Prenatal	0.62	0.82	0.28	0.28	0.36	0.43
	0-2 yr						
	20-35 yr						
	36-60 yr						
	>61 yr						
Frontal cortex	Prenatal	0.19	0.12	0.56	0.90	0.24	0.22
	0-2 yr						
	20-35 yr						
	36-60 yr						
	>61 yr						

yr: years
